# Alzheimer's Disease counteracted by Intravenous Antioxidants Bio supplements

**DOI:** 10.1002/alz70859_106341

**Published:** 2025-12-26

**Authors:** Juan A Moreira

**Affiliations:** ^1^ CNI‐ Centro Neurointegrativo, San Juan, Puerto Rico Puerto Rico

## Abstract

**Background:**

Alzheimer’s disease is the most common form of dementia, accounting for 60% to 70% of cases. (1) Current research indicates that oxidative stress is the principal cause of pathology linked to Alzheimer’s disease. (2‐6) Neuronal degeneration in this disorder is considered to be caused by the secondary effects of an increase in oxidation and lower mitochondrial bioenergetics with the lack of energy ending in neuronal death. (2‐6)

A reduction in ATP levels is caused by mitochondrial malfunctions that are characterized by decreased electron transport rates in complex I, III and IV of the mitochondrial respiration, increased oxidative stress – reactive oxidative species, decrease in glucose utilization associated to decrease activity and expression of Pyruvate Dehydrogenase Complex (PDH), elevation in glycolysis and loss in metabolic capabilities are early AD pathophysiological findings (2‐6)

Rationality: Infusion therapy of intravenous and/or intraspinal cranio‐cervical injections of antioxidant biosupplements (NAD, Alpha Lipoic Acid, Glutathione, Vitamin C) may serve to counteract the oxidation and inflammation that occurs in Alzheimer's disease by increasing the redox potential inside the mitochondria, which facilitate the production of neurotransmitters and reverse the loss of brain energy production at the chemical level, halting the cognitive deficit progression.

**Method:**

In the first month, fifty (50) patients received two initial biosupplements infusion treatments, which was then followed by a monthly infusion throughout the year. Intravenous biosupplements (NAD, Alpha Lipoic Acid, Vitamin C, and Glutathione) were administered. MOCA and ADAS‐ cog were measured at baseline and after one year receiving treatment.

**Result:**

Twenty‐seven (27) of the fifty patients showed no signs of deterioration, eighteen (18) showed mild improvement at the end of the year on MOCA and ADAS‐cog. Five (5) patients showed mild deterioration. No side effects were observed.

**Conclusion:**

Clinical symptoms of Alzheimer’s disease can be medically controlled with an appropriate intravenous bionutritional antioxidant combination as a treatment by improving the energy production of the brain. A clinical trial with a larger population should be considered.

